# Erfahrungen älterer, multimorbider Menschen in der COVID-19-Pandemie: eine qualitative Studie

**DOI:** 10.1007/s00391-022-02055-1

**Published:** 2022-04-06

**Authors:** F. H. Boehlen, M. K. P. Kusch, P. Reich, V. S. Wurmbach, H. M. Seidling, B. Wild

**Affiliations:** 1 Klinik für Allgemeine Innere Medizin und Psychosomatik, Universitätsklinikum Heidelberg, Im Neuenheimer Feld 410, 69120 Heidelberg, Deutschland; 2 Abteilung Klinische Pharmakologie und Pharmakoepidemiologie, Universitätsklinikum Heidelberg, Heidelberg, Deutschland; 3 Kooperationseinheit Klinische Pharmazie, Universitätsklinikum Heidelberg, Heidelberg, Deutschland

**Keywords:** COVID-19, Multimorbidität, Coping, Soziale Kontakte, Selbstwirksamkeit, COVID-19, Multimorbidity, Coping, Social contacts, Self-Efficacy

## Abstract

**Hintergrund/Ziel der Studie:**

Die COVID-19-Pandemie und die Maßnahmen zu deren Eindämmung haben das soziale Leben auf ungeahnte Weise verändert. Multimorbide, ältere Menschen, die ein hohes Risiko für schwerwiegende Krankheitsverläufe haben, wurden mit Nachdruck gebeten, Kontakte zu meiden, um das Infektionsrisiko zu mindern. Während dies von einem psychosozialen Standpunkt besorgniserregend wirkt, gibt es auch Hinweise, dass Ältere gelassener mit der Krise umgehen. Ziel der Studie war es, die Haltung von multimorbiden, älteren Menschen in der Pandemie zu beschreiben. Dazu wurden ihr Erleben, ihre sozialen Kontakte und die Erfahrungen mit medizinischer Versorgung ausgewertet.

**Material und Methoden:**

Zu 4 unterschiedlichen Zeitphasen (Juli 2020, September 2020, November 2020, Januar 2021) wurden halbstrukturierte Kurzinterviews zum Erleben der COVID-19-Pandemie mit multimorbiden, älteren Menschen in stationär-internistischer Behandlung geführt. Die Interviews wurden mithilfe der qualitativen Inhaltsanalyse analysiert.

**Ergebnisse:**

Es wurden die Daten von 21 Personen (Alter: 58 bis 88 Jahre) ausgewertet. Es wurde deutlich, dass die COVID-19 Pandemie – auch über die Zeit – sehr unterschiedlich erlebt wurde. Während in Phasen hoher Infektionsraten starke Affekte formuliert wurden, zeigte sich im Verlauf eine stärkere Differenzierung bis zur Verschiebung der Sorgen auf die gesellschaftliche Entwicklung. Das Verhältnis gegenüber Präventionsmaßnahmen oder der ärztlichen Versorgung war von Akzeptanz und Unterstützung geprägt.

**Diskussion:**

Diese Studie zeigt exemplarisch das Erleben älterer, multimorbider Menschen im Verlauf der Pandemie. Diese zeigen in vielen Bereichen ein hohes Maß an Anpassung und Reflexion sowie Akzeptanz der Umstände und sozialen Änderungen.

**Zusatzmaterial online:**

Zusätzliche Informationen sind in der Online-Version dieses Artikels (10.1007/s00391-022-02055-1) enthalten.

## Abstract (en)

**Background/objective:**

The COVID-19 pandemic and the accompanying preventive measures have shaped social life in unexpected ways. Because older persons with multiple chronic conditions have a high risk of a severe medical outcome, it has been strongly recommended that social contacts be curtailed in order to minimize risks of infection. While this appears to be alarming from a psychosocial point of view, it has been shown that older persons exhibit a high degree of equanimity and a good ability to cope with the crisis. The aim of the study was to describe the attitudes of multimorbid older people to the pandemic, their social contacts and their experiences with medical care.

**Material and methods:**

This cross-sectional qualitative survey was based on 21 semi-structured short interviews of older patients with multiple chronic conditions during inpatient health care, at 4 different points in time: July 2020, September 2020, November 2020 and January 2021. The data were analyzed by qualitative content analysis.

**Results:**

The statements of 21 participants (aged 58–88 years) were assessed. Over the course of the COVID-19 pandemic it became apparent that participants experienced the pandemic differently, both from an individual perspective and over time. While high infection rates were accompanied by serious concerns about health, periods of moderate infection risk were dominated by worry about social changes. In older persons there was a great sense of acceptance of the preventive measures.

**Conclusion:**

Our study exemplarily illustrates the attitudes and concerns of older persons who suffer from multiple chronic conditions over the course of the pandemic. Our data show that older persons reacted with equanimity to the novel medical and social circumstances.

Die COVID-19(Corona Virus Disease 2019)-Pandemie hat das Jahr 2020 geprägt. Das Virus und die Maßnahmen zur Bekämpfung haben zu neuen sozialen Strukturen und teilweise zu Veränderungen in der Gesundheitsversorgung geführt: Realitäten, die bewertet und verarbeitet werden mussten. Die vorliegende Arbeit gibt über eine qualitative Analyse Einblick in die Erfahrungen multimorbider älterer Menschen im zeitlichen Verlauf der Pandemie.

## Hintergrund und Ziel der Arbeit

Zu Beginn der COVID-19-Pandemie wurden ältere, multimorbide Menschen – Angehörige der sog. Risikogruppe – aufgefordert, soziale Kontakte und Tätigkeiten weitestgehend zu meiden. Dabei meint der Begriff „Risikogruppe“ Menschen, die „ein höheres Risiko für einen schweren Krankheitsverlauf (bei Infektion mit SARS-CoV-2) haben“ [13], Menschen älter als 50 Jahre und solche mit bestimmten Vorerkrankungen: z. B. Herz-Kreislauf- oder Krebserkrankungen. In der ersten Phase der Pandemie wurde zudem zeitweise der Regelbetrieb von Krankenhäusern und Arztpraxen eingeschränkt, um mehr Kapazitäten für die Versorgung von COVID-19-Patienten zu schaffen [4]. Es lässt sich vermuten, dass Veränderungen wie diese zu einer Zunahme von Ängsten und Einsamkeit bei älteren Menschen führen. Verschiedene Studien belegen eine enge Verbindung zwischen Einsamkeit und Isolation sowie dem Erleben von Unsicherheit mit psychischen Erkrankungen, auch im Rahmen der Pandemie [6, 7]. Dagegen sprechen andere Studien eher dafür, dass ältere Menschen mit einer gewissen Gelassenheit durch die Zeit der Krise kommen [2, 3, 14]. Hier ist anzunehmen, dass u. a. das Erleben von Selbstwirksamkeit eine mögliche Gelassenheit in der Krise moderiert [7]. Dabei wird Selbstwirksamkeit als Vertrauen in die eigenen Kompetenzen verstanden, als Überzeugung, schwierige Situationen erfolgreich bewältigen zu können [1]. Eine aktuelle Metaanalyse zeigt, dass sich ältere Menschen, die gesundheitliche Versorgung brauchen, als vermindert handlungsfähig empfinden [17] – was sich in der Pandemie in vermehrten Ängsten und Unsicherheit widerspiegeln könnte.

Ziel dieser Arbeit ist es herauszufinden, wie ältere Personen mit kritischen Vorerkrankungen die Pandemie wahrgenommen haben. Dabei wurden Schwerpunkte auf das affektive Erleben, die sozialen Kontakte und die Erfahrungen mit dem Gesundheitssystem gelegt.

## Studiendesign und Untersuchungsmethoden

### Datenerhebung.

Die Datenerhebung erfolgte über ein qualitatives Studiendesign. Der Fragebogen wurde durch die Studiengruppe, bestehend aus ÄrztInnen, PsychologInnen und PharmakologInnen, im Mai 2020 entwickelt, auf Basis einer Literaturrecherche in den Datenbanken PubMed und Google Scholar mit den Schlüsselbegriffen COVID-19, psychische Belastung, Ressourcen, soziale Isolation, Gesundheitsversorgung. Der finale Fragenkatalog bestand aus einem offenen Teil, in dem die spontanen Reaktionen zum Thema „Corona“ erfasst wurden, und einem spezifischen Teil, in dem das affektive Erleben, der Einfluss auf die sozialen Kontakte und die Erfahrungen im Gesundheitswesen erfragt wurden. Der Fragebogen wurde mit 3 PatientInnen pilotiert und dann, nach intensiver Diskussion in der interdisziplinären Expertenrunde, finalisiert. Die ersten Interviews – von Juli 2020 bis September 2020 – legten einen Fokus auf die erste Welle der Pandemie. Im Zuge der zweiten Welle wurden der Fragebogen im Oktober 2020 erweitert und das Erleben zu Beginn der Pandemie, aber auch in den vorangegangenen 4 Wochen erfasst. Appendix 1 (Zusatzmaterial online) gibt einen Überblick über die gestellten Fragen in den Phasen I und II. Alle Interviews wurden digital aufgezeichnet und nach wissenschaftlichen Standards transkribiert.

### Stichprobenrekrutierung und -beschreibung.

Die Datenerhebung fand im Kontext einer Studie statt, die die Prioritäten, Beschwerden und Ressourcen von älteren Menschen erfasst (PACT [19]). Eingeschlossen wurden Menschen mit mindestens 2 chronischen Erkrankungen ab 55 Jahren. Im Zeitraum von Juli 2020 bis Januar 2021 wurden mit allen Teilnehmenden zusätzlich begleitende Kurzinterviews zum Thema COVID-19 geführt. In diesen Studienteil wurden kontinuierlich 21 PatientInnen in stationärer internistischer Behandlung aufgenommen. Ausschlusskriterien waren unzureichende Deutschkenntnisse und schwergradige kognitive Defizite im ärztlichen Kontakt. Angepasst an den Verlauf der Pandemie wurden 4 Phasen – mit jeweils unterschiedlichen Personen – definiert. Aus Phase 1 wurden 6 Interviews eingeschlossen (aus insgesamt 9), in den Phasen 2, 3 und 4 wurden alle Interviews eingeschlossen (jeweils 5). In Phase 1 (t1; Juli 2020) war die Ausbreitung von SARS-CoV‑2 recht gering. In Phase 2 (t2; September 2020) nahmen die COVID-19-Erkrankungen wieder langsam zu. Phase 3 (t3; 15.11.2020–15.12.2020) umfasste den „Lockdown light“ mit hohen Infektionszahlen, und Phase 4 (t4; 15.12.2020–31.01.2021) fiel in die Zeit des zweiten „harten Lockdowns“, in dem die Infektionszahlen unverändert hoch waren, aber auch die Impfungen gegen COVID-19 begannen.

### Erhebung demografischer und klinischer Variablen.

Begleitend zu den Interviews wurden durch den Studienmitarbeiter klinische Daten erhoben. Diese umfassten demografische Angaben, die Diagnosen nach ICD-10 und die Medikation. Depressive Symptome wurden über den Score des Patient Health Questionnaire (PHQ-9) [8] erfasst (leicht: 5–9/mittelgradig: 10–14/schwer: 15–27). Alle Teilnehmenden erhielten das INTERMED-Interview zur Einschätzung ihres Gesundheitszustands [5]. Ab einem INTERMED-Score von 21 wird von komplexem Versorgungsbedarf gesprochen, bei dem ein hohes Risiko für schwere Krankheitsverläufe vorliegt. Die gesamte Datenerfassung nahm ca. 60 min in Anspruch, davon entfielen 2–6 min auf die Kurzinterviews zum Thema COVID-19.

### Auswertung.

Die Transkripte wurden mithilfe der qualitativen Inhaltsanalyse nach Mayring analysiert [10]. Erhebung und Auswertung wurden im Wechsel durchgeführt, bis im Sinne einer theoretischen Sättigung keine neuen Erkenntnisse auftraten [15]. Über einen deduktiven-induktiven Prozess wurden Haupt- und Unterkategorien durch 2 unabhängige Rater gebildet. Die spontanen Äußerungen bezüglich Corona wurden induktiv kategorisiert und hinsichtlich der zeitlichen Dynamik betrachtet. Die spezifischen Themenfelder (Affekte, soziale Auswirkungen, Erleben des Gesundheitswesens) wurden deduktiv-induktiv analysiert. Der Analyseprozess wurde regelmäßig in der Forschungsgruppe diskutiert und konsentiert. Die Datenanalyse erfolgte mit MaxQDA, Version 2020 (VERBI GmbH, Berlin, Deutschland).

## Ergebnisse

Eine Übersicht über die Charakteristika der 21 TeilnehmerInnen gibt Tab. 1. Angaben zu den Untergruppen finden sich in Appendix 2 (Zusatzmaterial online). Es fiel auf, dass die depressive Beschwerdelast in den Phasen 3 und 4 (November 2020 bis Januar 2021) deutlich höher lag als in den Phasen 1 und 2 (07–09/20) (PHQ- Score: 11,1 ± 5,6 vs. 6,5 ± 4,7). Alle Interviewten befanden sich aufgrund eines akuten kardialen Ereignisses in stationärer Behandlung.

**Table 1:** 

Anzahl Interviews	21 (11 weiblich, 10 männlich) (t1:6; t2/t3/t4:5)
Alter	69,3 ± 8,2 Jahre; Min.: 58; Max.: 88
Familienstand	12 verheiratet, 1 alleinstehend, 6 geschieden/verwitwet, 20 zu Hause lebend, 1 im Pflegeheim
Bildungsstand	10 Volksschule, 3 Realschule, 3 Abitur/Hochschulbildung, 4 Sonstige
Anzahl, Diagnosen	5,3 ± 2,9; Min.: 2; Max.: 17
Häufigste Diagnosen	Herzrhythmusstörungen, KHK, Herzklappenvitien, Kardiomyopathie pAVK, Niereninsuffizienz, Schlafapnoe, Anpassungsstörung
Anzahl, Medikamente	5,9 ± 3,5; Min.: 0; Max.: 21
Depressionssymptome	PHQ-9: Leicht: 6/Mittelgradig: 4/Schwer: 4; k. A.: 1 Mittelwert: 7,6 ± 5,4; Range 0–27
INTERMED-Interview	Mittelwert: 24,5 ± 6,6; 17 komplex (81 %), Range: 0–60

### Erfahrungen mit und Auswirkungen der COVID-19-Pandemie.

**Figure 1:**
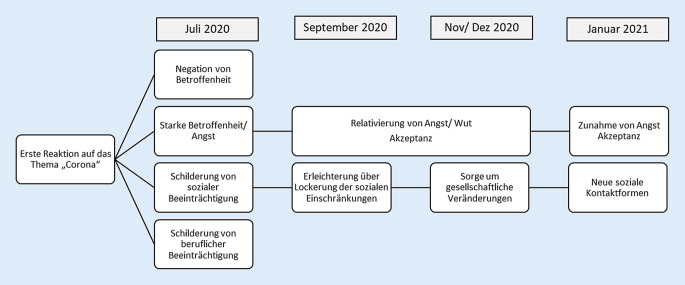

Direkt auf das Thema „Corona“ und die Erfahrungen im Rahmen der ersten Welle angesprochen, wurden einerseits affektive Betroffenheit thematisiert und andererseits die sozialen Auswirkungen der Pandemie (Abb. 1).

Im Juli 2020 zeigten sich Reaktionen, die von Negation jeglicher Betroffenheit bis hin zu starker Angst reichten. Dabei fiel auf, dass das Ausmaß der Betroffenheit unabhängig davon war, wie stark die PatientInnen vorerkrankt waren. Von den 6 beteiligten Personen zeigten sich eher die Personen, die noch berufstätig waren, eingeschränkt und besorgt.

*„Corona* *…. Habe ich nichts mit zu tun“*

(TN 1.1: weiblich, über 80 J., verwitwet, alleinlebend, berentet, komplex erkrankt, leicht depressiv)

*„Einschränkungen …Ja also erstmal im Geschäft, ist ja logisch … Ich hatte ja fast drei Monate zu …. man wusste ja auch nicht, wie gestaltet sich da die Zukunft, ja. Muss ich mein Geschäft ganz zumachen, oder wie geht es überhaupt weiter?“*

(TN 1.6: weiblich, 70–80 J., verheiratet, berufstätig, komplex erkrankt, keine psychische Erkrankung)

In den Gesprächen im September 2020 wurde der Blick auf die erste Welle der Pandemie eher relativierender. In verschiedenen Gesprächen war Erleichterung über die Lockerungen der sozialen Beschränkungen und die Erweiterung des eigenen Handlungsspielraums zu spüren.

*„Am Anfang hatte ich große Angst, …, aber dann haben wir uns alle mal unterhalten. Und dann haben wir gesagt, also, theoretisch kann uns ja nichts passieren. Wir halten ja alles ein … wir halten Abstand, wir tragen Mundschutz. Wir verhalten uns so, wie man sich verhalten soll, machen alles richtig. Und jetzt sage ich mir: Also, wenn ich es kriege, dann kriege ich es.“*

(TN 2.1: weiblich, 60–70 J., verheiratet, berufstätig, komplex erkrankt, mittelgradig depressiv)

In den Interviews im November 2020 wurden zunehmend Sorgen über die gesellschaftlichen Auswirkungen deutlich, bzw. auch Ärger über das Nichteinhalten von Schutzmaßnahmen.

*„Weil halt viele mittlerweile halt in ihrer Spaßgesellschaft eingeschränkt sind. Und irgendwie nicht akzeptieren können, dass es einfach, dass man sich mal zurücknehmen muss, ja. … Die Älteren vielleicht besser, weil die noch den Krieg erlebt haben, und da war halt immer was nicht da oder ging was nicht.“*

(TN: 3.1: weiblich, über 80 J., verwitwet, alleinlebend, berentet, nicht komplex erkrankt, mittelgradig depressiv)

Im Dezember 2020/Januar 2021 wurden in den Gesprächen spontan weniger die Restriktionen im Rahmen der Pandemie diskutiert, sondern wieder mehr die Erkrankung selbst. Dabei zeigten die PatientInnen erneut eine Zunahme von Ängsten in Bezug auf COVID-19.

### Spezifische Themenfelder zum Thema COVID-19.

**Figure 2:**
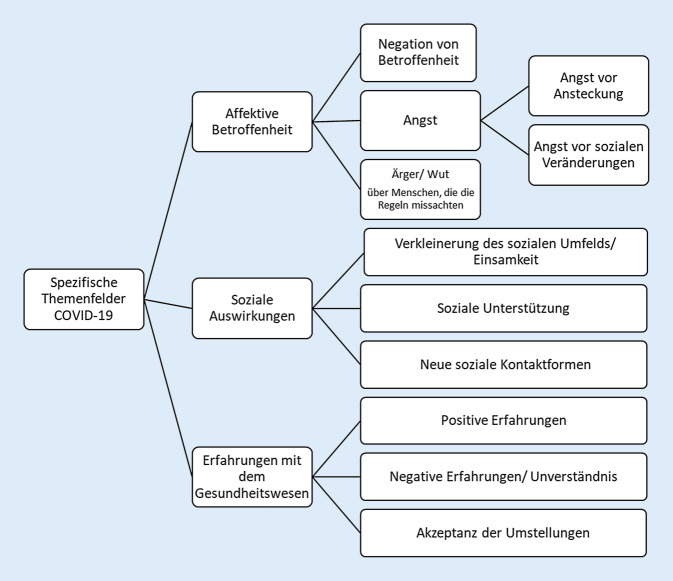

Im zweiten Teil des Interviews wurden die affektive Betroffenheit, die sozialen Änderungen und die Erfahrungen mit dem Gesundheitssystem erfragt. Affektiv wurden Angst und Wut, aber auch eine Negation von Betroffenheit deutlich. Hinsichtlich der sozialen Auswirkung wurde einerseits die Verkleinerung des sozialen Umfelds beschrieben, aber auch die Art der Unterstützung und neue Formen des Kontakts. Die Änderungen im Gesundheitswesen wurden positiv, neutral bis negativ eingeordnet (Abb. 2).

### Gefühlslage der Teilnehmenden.

Die affektive Betroffenheit zeigte sich interindividuell schwankend. Am häufigsten wurden Emotionen wie Angst und Wut genannt, die mit dem Fortschreiten der Pandemie reflektierend eingeordnet wurden. Im November 2020 wurde dann erstmalig begleitend zur Angst auch Ärger geschildert, der sich jedoch auf die Nichteinhaltung von Schutzmaßnahmen richtete.

*„Von den Symptomen, die da immer rumgegeistert sind, fand ich dann immer wieder welche bei mir … Je nachdem, was blüht. … Bis man das dann sortiert kriegt und sicher ist, das ist halt nicht Corona. Das hat eine Weile gedauert.“ (September 2020)*

(TN 2.4: männlich, 60–70 J., Familienstand/Berufsstand: n. b., komplex erkrankt, leicht depressiv)

*„Man hat schon ein bisschen Angst. Ah ja, wem macht das keine, also, ja doch, es gibt so ein paar Idioten, die, ja, die Leute verstehe ich nicht.“ (Dezember 2020)*

(TN 3.3: weiblich, 60–70 J., verheiratet, berentet, komplex erkrankt, keine psychische Erkrankung)

### Soziale Auswirkungen.

Es traten 3 unterschiedliche Themenfelder auf: die sozialen Einschränkungen, die dynamischen Änderungen des sozialen Umfelds und die soziale Unterstützung. Diese wurde nur im Einzelfall über Sozialstationen geleistet, sondern primär über die Kernfamilie. Auch schilderten einzelne, dass der Kontakt zu den Enkeln gehalten wurde, wenn auch mit physischer Distanz. Andere schränkten sich gerade in der Anfangszeit der Pandemie sehr ein und gingen insbesondere nicht mehr zum Einkaufen. Auch das Thema Einsamkeit wurde erwähnt, jedoch eher nüchtern einordnend. Im Jahresverlauf zeigte sich eine hohe Akzeptanz der Maßnahmen im sozialen Bereich bis hin zur Erprobung neuer sozialer Kontaktformen.

*„Aber ab und zu bin ich auch gegangen, aber ich gehe so gut wie nicht mehr. Mein Mann will das auch nicht, weil er sagt: Du bist gefährdeter als ich und du bleibst zu Hause. Oder ganz schlimm war ja im März, April, dass die Familie nicht kommen durfte. Sie glauben nicht, unsere Enkelkinder, als wir uns zum ersten Mal wiedersehen durften und in den Arm nehmen, die habe uns nicht mehr losgelassen.“ *(November 2020)

(TN 3.3.: weiblich, 60–70 J., verheiratet, berentet, komplex erkrankt, keine psychische Erkrankung)

*„Und wir hatten Urlaub geplant, mit Freunden, das ist alles ausgefallen dieses Jahr, das ist natürlich klar. Aber sagen wir es mal so; es belastet mich nicht. Das nimmt man hin, und nächstes Jahr oder so versuchen wir es nochmal.“ (Januar 2021)*

(TN 4.4: männlich, 60–70 J., verheiratet, komplex erkrankt, mittelgradig depressiv)

*„Was heißt einsamer. Eigentlich nicht. Ich bin dadurch, dass ich jetzt schon so lange alleine lebe, bin ich das irgendwie gewohnt, ja.“*

(TN: 3.1: weiblich, über 80 J., verwitwet, alleinlebend, berentet, nicht-komplex erkrankt, mittelgradig depressiv)

### Wahrnehmung der Gesundheitsversorgung.

Hinsichtlich der Erfahrungen mit der Gesundheitsversorgung skizzierten die TeilnehmerInnen zu allen Phasen der Pandemie ein ausgewogenes Bild. Während Einzelne auf Termine warten mussten oder Unmut äußerten, zeigte der Großteil Verständnis für die Anpassungsleistungen der Versorger oder äußerten sich positiv über das Engagement.

*I: „Also das Hauptproblem war ja, dass Termine abgesagt worden sind.“ TN: „Nein, eigentlich nicht. Im Gegenteil, man kriegt mehr Zeit. Weil es besser koordiniert ist.“ I: „Und hier im Krankenhaus, hatten Sie irgendwelche Ängste oder …“ TN: „Eigentlich nicht. Angenehm, wenn nicht so viel los ist.“ (November 2020)*

(TN 3.2: männlich, 60–70 J., ledig, berentet, komplex erkrankt, keine Angabe zu psychischen Erkrankungen)

*„Dann geht das sofort. Da hat sich auch mit Corona nichts verändert.“ (Januar 2021)*

(TN 4.1: männlich, 60–70 J., verheiratet, berentet, komplex erkrankt, leicht depressiv)

Im Vergleich von Frauen und Männern zeigten sich nur geringe Unterschiede in den emotionalen Äußerungen. Es fiel jedoch auf, dass Frauen bei der Schilderung ihres sozialen Netzes eher Bezug auf die Kinder und Enkel nahmen, während Männer eher die Änderungen im Freundeskreis erwähnten. In der Untergruppe der PatientInnen mit mittel- bis schwergradiger depressiver Symptomatik (*n* = 8) zeigte sich ein breites Spektrum. Während 5 Personen die sozialen Einschränkungen als belastend beschrieben, gaben zwei an, von der Kontaktreduktion durch den Wegfall von Verpflichtungen zu profitieren.

Die durchschnittliche Länge der auf „COVID-19“ bezogenen Interviews betrug 3:32 min. Die Länge der Interviews nahm im Laufe der Zeit zu (2:33 min im Juli 2020 bis 5:28 min im Januar 2021).

## Diskussion

Unsere Studie zeigt an exemplarischen Fällen die Erfahrungen älterer, multimorbider Menschen in akut-medizinischer Behandlung zu verschiedenen Phasen der Pandemie. Sie spiegelt den Verlauf der Pandemie im affektiven Erleben sowie in der Schilderung des Adaptationsprozesses an ein verändertes soziales Gefüge. Dabei zeigte sich in vielen Bereichen ein hohes Maß an Pragmatismus und Reflexion sowie Akzeptanz.

Erwartbare Emotionen gegenüber COVID-19, wie Angst oder Verunsicherung, werden genannt, dies jedoch unabhängig davon, wie schwer erkrankt die einzelne Person ist und welchem Geschlecht sie angehört. Die 70-jährige, verhältnismäßig gesunde Patientin, die eine hohe Beeinträchtigung, bedingt durch Veränderungen im beruflichen Umfeld beschreibt, gehört ebenso zur Gruppe der vulnerablen älteren Personen, wie die schwer multimorbide 75-Jährige, die eine Betroffenheit schlicht verneint. Eine vergleichbare Heterogenität findet sich in der Literatur. Während einige Autoren die psychische Resilienz älterer Menschen in der Coronakrise herausstellen und keine signifikante Zunahme an Einsamkeit beobachten [9], warnen andere, dass die pandemiebedingte Isolation durchaus auch traumatisierend verarbeitet werden kann [16]. Beide Beobachtungen finden Wiederklang in unseren Daten. Dabei ist auffällig, dass Ängste zunächst eher verneint, dann relativiert werden und im Zuge der zweiten „Corona-Welle“ wieder zunehmen. Eine ähnliche Dynamik findet sich in anderen Studien, die die größte Belastung durch die Pandemie in Phasen der größten Bedrohung sehen [7]. Auch werden Gefühle wie Ärger thematisiert, jedoch eher gegenüber denen, die sich nicht an die Schutzmaßnahmen halten.

Die TeilnehmerInnen unserer Studie weisen eine überdurchschnittlich hohe Morbidität und Depressivität auf, im Vergleich mit anderen Gruppen ähnlichen Alters [18] – bedingt durch die Einschlusskriterien der Studie und die Rekrutierung im stationären Umfeld. Trotzdem scheinen sie sorgsam, aber gelassen angesichts COVID-19. Diese Gelassenheit könnte auf der Lebenserfahrung und dem Wissen um den Umgang mit potenziell lebensbedrohlichen Situationen oder sozialen Änderungen beruhen. Es ist aber auch möglich, dass das soziale Umfeld im Vorfeld schon so konzentriert war, dass die Änderungen nicht gravierend schienen. Bezogen auf das Konzept der Selbstwirksamkeit wird bei einigen TeilnehmerInnen deutlich, dass ihre Zuversicht wuchs, durch die zunehmende Erfahrung mit der Pandemie und die Überzeugung, diese Situation auch selber bewältigen zu können. Dabei fällt auf, dass viele Interviewte häufiger die eigene Handlungskompetenz gegenüber COVID-19 herausstellten, z. B. über Schutzmaßnahmen oder neue Kontaktformen, und weniger auf die mögliche Bedrohung angesichts der eigenen Vorerkrankung eingingen.

Hinsichtlich der Auswirkung von depressiver Symptomatik auf das Erleben der Pandemie zeigte sich in unseren Daten keine klare Tendenz. Weder zeigten depressive TeilnehmerInnen ein ausgeprägteres Rückzugverhalten noch eine auffallend passiv-pessimistische Haltung gegenüber COVID-19. Es fiel aber auf, dass die TeilnehmerInnen aus den späteren Phasen eine höhere depressive Symptomatik zeigten. Dabei lässt sich jedoch nicht sagen, ob dies durch die fortdauernde Pandemie zu erklären ist, oder ob saisonale Effekte eine Rolle spielen.

Auf sozialer Ebene fiel auf, dass die Änderungen, insbesondere von aktiven Älteren bedauert, jedoch weitestgehend akzeptiert wurden. Dabei schilderten einige, dass sich durch COVID-19 der Kontakt zur direkten Familie änderte, räumlich nahe Kontakte sogar z. T. intensiviert wurden. Andere schilderten die Aufnahme neuer Kontaktmodelle, wie Videochats, gaben aber auch an, dass diese den Kontakt nicht vollständig ersetzten. In diesem Kontext zeigte ein aktueller Review, dass es bislang keine gesicherte Evidenz gibt, dass Videotelefonate psychische Belastung älterer Menschen signifikant lindern [11]. Weiterhin war auffällig, dass die Alltagsunterstützung der befragten „Risikopatienten“ primär von familiären Bezugspersonen vorgenommen wurde und weniger von Sozialdiensten. Dies wurde auch in anderen Studien beschrieben, die im Rahmen der COVID-19-Pandemie eine Zunahme der Versorgungsarbeit in den Familien, insbesondere bei Frauen, sahen [12].

Hinsichtlich der medizinischen Versorgung fiel eine hohe Zufriedenheit auf, sowie Verständnis für die getroffenen Schutzmaßnahmen oder die Aufschiebung von Terminen. Dabei wurde die Ansprechbarkeit der HausärztInnen hervorgehoben. Von Einzelnen wurde der erhöhte organisatorische Vorlauf, bedingt durch die Schutzmaßnahmen, als strukturierend eingeordnet.

Unsere Ergebnisse sind von Limitationen betroffen. So unterliegen die Aussagen zum zeitlichen Verlauf deutlicher interindividueller Schwankung, da sich jede Teilkohorte aus unterschiedlichen Menschen zusammensetzt und so pro Zeitraum jeweils nur sehr individuelle Meinungen erfasst wurden. Auch wurden wichtige Aspekte wie der soziale Status und die soziale Situation vor der Pandemie nicht detailliert erfasst. Auch ist es möglich, dass durch die kurze Dauer dieses Interviewabschnitts belastete Themenfelder bezogen auf COVID-19 nicht ausreichend thematisiert werden konnten. Weiterhin ist auffällig, dass die Länge der Interviews zunahm. Dies kann einerseits an der geschilderten Modifikation des Fragebogens liegen, aber auch an der zunehmenden Erfahrung mit COVID-19 im Zuge der immer länger andauernden Pandemie. Die größte Stärke hingegen ist die Erhebung von Daten in einer Gruppe von hochbelasteten PatientInnen, die in vielen aktuellen Studien zu COVID-19 nicht abgebildet sind.

Dies berücksichtigend stellen unsere Daten wichtige Momentaufnahmen aus dem Erleben der COVID-19-Pandemie von älteren Menschen mit kritischen Vorerkrankungen dar, aus denen sich Ansatzpunkte für weitere Forschungsarbeiten ergeben können. Dabei ist jedoch auch weiterhin die unverändert hohe Dynamik der Pandemie zu beachten.

## Fazit für die Praxis

- Es ist auffällig, dass in der Gruppe der befragten PatientInnen viele der Krise mit Gelassenheit und Pragmatismus begegnen.
- Ängste (vor Ansteckung oder vor sozialen Veränderungen) schienen im Laufe der Pandemie zunächst abzunehmen, im Rahmen der zweiten Infektionswelle jedoch wieder anzusteigen.
- Ältere multimorbide Menschen schildern eher unveränderte, z. T. sogar positive Erfahrungen hinsichtlich ihrer medizinischen Versorgung.

## Supplementary Information





