# Supplementary material for: Experiences of older multimorbid persons during the COVID-19 pandemic: a qualitative study
Source: Z Gerontol Geriatr. 2022 Apr 6;55(3):216–22. [Article in German] doi: 10.1007/s00391-022-02055-1 (PMC8984670; doi:10.1007/s00391-022-02055-1)
Supplement: Supplementary file 1 [file 391_2022_2055_MOESM1_ESM.docx]

Appendix 1: Fragenkatalog zur COVID-19- Pandemie

| Einstieg | |
| --- | --- |
| In den letzten Monaten hat COVID-19 das Leben von uns allen beeinflusst. Was hat sich denn bei Ihnen verändert seit dem Beginn der Pandemie? Was würden Sie denn sagen war vor der Pandemie besser? Und was war schlechter vor Corona? – *Version 2 Ergänzung: Hat sich Ihre Einstellung zu COVID-19 in den letzten 4 Wochen geändert?* | |
| Spezifische Fragen | |
| Gefühlslage | Wie geht es Ihnen, wenn Sie an COVID-19 denken?Wie würden Sie ihre allgemeine Gefühlslage während der bisherigen Zeit beschreiben? Gibt es Veränderungen zu früher?  *Version 2 Ergänzung: Haben sich Ihre Gefühle in den letzten 4 Wochen geändert? Wie blicken Sie in die Zukunft?* |
| Soziale Kontakte | Zu wem hatten Sie während dieser Zeit Kontakt und wie hatten Sie Kontakt? (ggf. Nachfrage: Hatten Sie noch andere Kontakte, zum Beispiel zur Unterstützung? Wie haben sich ihre Kontakte verändert? Wenn Sie an die Zeit vor COVID-19 zurückdenken, haben Sie sich damals öfter einsam gefühlt? Und wie ist das heute? Fühlen Sie sich eher einsamer als vorher oder eher weniger einsam? |
| Gesundheit/ Gesundheitssystem | Hat sich ihr Kontakt zu Ärzten/ Therapeuten verändert? Wenn ja, wie? Wie fühlen Sie sich denn vor dem Hintergrund der aktuellen Situation in unserem Gesundheitssystem? Fühlen Sie sich gut versorgt? |
| Informationsverarbeitung &-gewinnung | Wie geht es Ihnen mit den Informationen, die Sie über COVID-19 lesen oder hören? Woher bekommen Sie denn Ihre Informationen hauptsächlich (z.B. aus dem Internet)? *Version 2 Ergänzung: Hat sich Ihre Einstellungen gegenüber Informationen verändert?* |
| Abschluss | |
| Wir haben jetzt über verschiedene Bereiche in ihrem Leben gesprochen, z.B. Ihre sozialen Kontakte, Ihr Gefühlsleben und die Gesundheitsversorgung. In welchem Bereich merken Sie die Veränderungen durch COVID-19 denn am stärksten?  Was tut Ihnen momentan gut, zum Beispiel in Bezug auf Ihre Gesundheitsversorgung ? Und fehlt Ihnen etwas? Gibt es vielleicht etwas, dass Sie in der aktuellen Situation besonders brauchen würden? | |

Version 1 vom 25.05.2020/ *Version 2: Anpassung vom 15.10.2020*
