# Supplementary material for: Experiences of older multimorbid persons during the COVID-19 pandemic: a qualitative study
Source: Z Gerontol Geriatr. 2022 Apr 6;55(3):216–22. [Article in German] doi: 10.1007/s00391-022-02055-1 (PMC8984670; doi:10.1007/s00391-022-02055-1)
Supplement: Supplementary file 2 [file 391_2022_2055_MOESM2_ESM.docx]

Appendix 2:

Demographische und klinische Daten der Studiengruppe- aufgeteilt nach den einzelnen Gruppen

**Phase 1: Juli 2020**

| Anzahl Interviews | 6 (3 weiblich, 3 männlich) |
| --- | --- |
| Alter | 71,7 Jahre (SD: 9,0) Min.: 64; Max.: 83 |
| Familienstand | 4 verheiratet, 2 geschieden/ verwitwet |
| Bildungsstand | 1 Volksschule, 2 Abitur/Hochschulbildung, 3 Sonstige |
| Anzahl Diagnosen | 6,8 (SD: 3,5) Min.: 3; Max.: 11 |
| Depressionssymptome | Mittelwert: 6,3 (SD: 6,2) |
| INTERMED- Interview | Mittelwert: 25,3 (SD: 6,7); 5 komplex |

**Phase 2: September 2020**

| Anzahl Interviews | 5 (4 weiblich, 1 männlich) |
| --- | --- |
| Alter | 69,0 Jahre (SD: 5,8) Min.: 62; Max.: 78 |
| Familienstand | 2 verheiratet, 2 geschieden/ verwitwet, 1 k. A. |
| Bildungsstand | 3 Volksschule, 1 Realschule, 1 k. A. |
| Anzahl Diagnosen | 7,0 (SD: 31,6) Min.: 5; Max.: 9 |
| Depressionssymptome | Mittelwert: 6,6 (SD: 1,6) |
| INTERMED- Interview | Mittelwert: 23,6 (SD: 8,4); 4 komplex |

**Phase 3: November/ Dezember 2020**

| Anzahl Interviews | 5 (3 weiblich, 2 männlich) |
| --- | --- |
| Alter | 68,4 Jahre (SD: 7,1) Min.: 61; Max.: 80 |
| Familienstand | 3 verheiratet, 1 geschieden/ verwitwet, 1 ledig |
| Bildungsstand | 2 Volksschule, 2 Realschule, 1 Abitur/Hochschulbildung |
| Anzahl Diagnosen | 9,0 (SD: 5,9) Min.: 5; Max.: 17 |
| Depressionssymptome | Mittelwert: 10,5 (SD: 7,1) |
| INTERMED- Interview | Mittelwert: 24,4 (SD: 6,2); 4 komplex |

**Phase 4: Dezember 2020/ Januar 2021**

| Anzahl Interviews | 5 (1 weiblich, 4 männlich) |
| --- | --- |
| Alter | 68,2 Jahre (SD: 11,4) Min.: 61; Max.: 88 |
| Familienstand | 2 verheiratet, 2 k. A. |
| Bildungsstand | 3 Volksschule, 1 Sonstige, 1 k. A. |
| Anzahl Diagnosen | 8,8 (SD: 4,7) Min.: 2; Max.: 13 |
| Depressionssymptome | Mittelwert: 11,6 (SD: 4,9) |
| INTERMED- Interview | Mittelwert: 24,8 (SD: 6,5); 4 komplex |
